# Community‐level wastewater surveillance with machine learning methods to assess underreporting of COVID‐19 case counts

**DOI:** 10.1002/mlf2.70055

**Published:** 2025-12-03

**Authors:** Nathan Szeto, Jianfeng Wu, Yili Wang, Xin Li, Zheshi Zheng, Leyao Zhang, Richard Neitzel, Marisa Eisenberg, J. Tim Dvonch, Alfred Franzblau, Peter X. K. Song, Chuanwu Xi

**Affiliations:** ^1^ Department of Biostatistics University of Michigan School of Public Health Ann Arbor Michigan USA; ^2^ Department of Environmental Health Sciences University of Michigan School of Public Health Ann Arbor Michigan USA; ^3^ Department of Epidemiology University of Michigan School of Public Health Ann Arbor Michigan USA; ^4^ Present address: Department of Industrial and Operations Engineering University of Michigan College of Engineering Ann Arbor Michigan USA

## Abstract

COVID‐19 remains an ongoing threat to public health, and reliable, continuous disease monitoring programs are essential for preventing future surges of infection. However, without mandated COVID‐19 testing, accurate data of confirmed cases are unavailable. Instead, COVID‐19 viruses may be tracked via wastewater samples from sewage manholes in areas of high social connectivity, where captured viral RNA data are biomarkers useful for monitoring and predicting community‐level COVID‐19 prevalence through machine learning techniques. We construct a prediction model of high sensitivity and specificity to provide evidence of significant underreporting of COVID‐19 cases for the time period following the lifting of testing mandates.

Despite a recent decline in concern regarding the COVID‐19 pandemic, the COVID‐19 virus continues to pose a significant threat to global public health. Even though 70% of the global population has received at least one dose of a COVID‐19 vaccine^1^, and prevailing SARS‐CoV‐2 variants exhibit reduced pathogenicity compared to the Delta variant^2^, ^3^, effective infectious disease monitoring remains essential. Therefore, effective infectious disease surveillance remains essential for tracking and controlling future disease outbreaks.

Traditionally, monitoring of infectious disease outbreaks has relied on in‐person clinical diagnostic testing. However, this method only captures a small fraction of actual infections^4^. Underestimation, including both under‐ascertainment and underreporting, of infection cases is extensively studied. Underreporting may arise from patient misdiagnoses attributable to the use of unreportable at‐home tests, atypical symptoms, limited test sensitivity, or a lack of strict diagnostic criteria^5^, ^6^, ^7^. In other words, underreporting refers to a data collection that inaccurately records the true number of disease cases by certain disease surveillance programs. Effective pandemic preparedness depends on an accurate estimate of the true infection case counts in a population. The underestimation of true infection case counts poses a major challenge in the monitoring of disease dynamics and analysis of epidemiological characteristics, which may result in inappropriate prevention and control policies.

Most previous studies on COVID‐19 have been based largely on Electronic Health Records (EHR) databases which include confirmed cases, hospitalized cases, and deaths in health care systems. Our data on daily confirmed cases were collected from University of Michigan (U‐M) hospital databases. Such databases may be susceptible to systemic deviations between reported and actual COVID‐19 case count data, potentially limiting their reliability. Unlike clinically‐captured data, wastewater‐based surveillance (WBS) provides a novel approach to address the challenge of underreporting. The SARS‐CoV‐2 virus is shed from the body primarily through feces containing virus RNA, which subsequently enters sewage systems. Pathogen‐shedding usually precedes symptom onset, healthcare‐seeking behavior, and subsequent diagnostic testing. Therefore, WBS methods can detect undiagnosed infections (including presymptomatic and asymptomatic cases), increases in pathogen loads, and impending increases in infected case counts^8^, ^9^. In addition, the ease with which pooled wastewater samples may be collected further improves the advantages of WBS methods. For example, they may be used to monitor large populations efficiently or to enable public health officials to monitor disease spread in real‐time^10^, ^11^. While WBS methods have been applied and validated in many studies, few have focused on university communities which are deemed as vibrant communities with high risk for disease transmission.

In this study, based on U‐M sewage water monitoring program and U‐M COVID‐19 case count data, the aim was to develop a machine learning prediction model to forecast COVID‐19 case counts during periods in which underreporting is suspected to be severe, primarily those following the broad lifting of mandatory testing requirements. Machine learning models have already been used to predict future case counts and estimate underestimation severity^12^. We use such a model to generate case count predictions, which are then compared to reported counts over the same time periods.

Specifically, we constructed a partial least squares regression (PLSR) model which yielded an *R*‐squared value of 0.425 for in‐sample predictions, indicating a satisfactory goodness of fit. Such in‐sample prediction performance lies in contrast to the out‐of‐sample predictions considered below, for which we want to demonstrate systemic underreporting and hence “poor” prediction fit. An *R*‐squared value of 0.425 is generally considered satisfactory for demonstrating reasonable goodness of fit, particularly given the weak signal provided by RNA concentration data. Furthermore, Figure S1 shows the predicted COVID‐19 case counts based on the U‐M data before May 2022 and the confirmed case counts by the university health system. Both time series of daily counts adhere closely with each other, suggesting that the model accurately predicts the COVID cases during the time when mandatory COVID testing was effective on the campus. See Materials and Methods in Supporting Information for more details.

The PLSR prediction model was then employed to predict the university‐level case counts after May 2022 following the broadly lifting of mandatory testing requirements. The predicted and reported case counts diverge, with university‐level predicted case counts significantly outpacing reported ones (Figure S2). These results suggest that severe COVID‐19 case underreporting is present after May 2022. It was found that during the period from May 2022 to April 2023, reported case counts could underreport true case counts at levels ranging from −43.50% to 81.98%. We determined that the average level of estimated underreporting was 47.16% over this time period. Instances of underprediction were present, where underprediction refers to the difference in which the daily predicted case counts were lower than daily reported ones from the university EHR database. Specifically, we observed 20 (or 8%) underprediction cases versus 277 (or 92%) underreporting cases. The key takeaway from Figure 1 is that underreporting occurs at an excessively higher frequency than underprediction, suggesting strong evidence that underreporting of COVID‐19 case counts post‐May 2022 in the university community is widespread. Furthermore, conformal prediction intervals (CPIs) are provided in Figure S3 to reflect prediction accuracy. Reported cases close to the lower bound of the 95% CPIs suggest further evidence of severe underreporting of true case counts.

**Figure 1:**
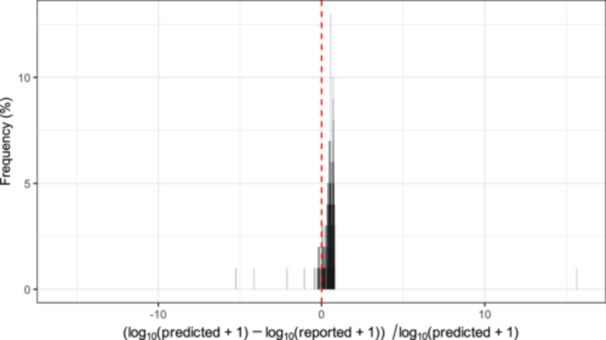
Distribution for the percent of underreporting COVID‐19 cases post‐May 2022 based on log_10_‐transformed predicted values. Negative values correspond to underprediction, while positive values refer to underreporting. To ensure legibility, one extremely negative value (−136.54) outside of two standard errors was excluded from this plot. This excluded value is likely to reflect model error rather than biologically pertinent information. Most importantly, a large majority of observed values correspond to underreporting, suggesting widespread underreporting in the data. The standard error is 7.924 and the interquartile range (IQR) is 0.347.

In this university‐community wastewater epidemiological study, evidence of severe underreporting (47.16% on average) of daily COVID‐19 case counts after May 2022 at the University of Michigan was observed. This underreporting is likely a result of the lifting of mandatory testing measures that occurred around this time. Based on the wastewater‐based RNA data, we built a machine learning prediction model based on presumably accurate data captured during periods of mandatory COVID‐19 testing required by the university to predict expected daily case counts after May 2022, when mandatory testing requirements were lifted. This approach enabled us to quantitatively demonstrate discrepancies between community‐level predicted and reported case counts post‐May 2022. Our findings are consistent with existing literature concerning systemic underreporting of COVID‐19 cases collected after mandatory testing policies were relaxed^6^, ^7^, ^13^, ^14^.

Our new contribution lies in the development of an accurate and robust prediction model using wastewater RNA concentration data. This model enables us to rigorously quantify the degree of underreporting severity, suggesting data quality issues in disease surveillance programs. Previous studies have shown that wastewater‐based surveillance is an effective method by which to track the spread of SARS‐CoV‐2, based on the premise that increases in SARS‐CoV‐2 RNA concentrations in sewage water are positively associated with case counts^8^, ^9^, ^10^, ^11^, ^12^, ^13^. The WBS approach demonstrated here advances data science techniques for the study of underreporting phenomena, and Figures 1 and S1, S2 provide evidence supporting further research of underreporting phenomena. This time‐stratified pre‐post study design has resulted in a useful analytic toolbox that may be applied to analyze similar data in wastewater epidemiology and more generally in infectious disease control. Moreover, the results in this study suggest that even in a high‐transmission communities, WBS is an effective approach for disease surveillance.

The uncertainty of the prediction model was quantified to ensure that the influence of the sampling randomness on the prediction model was properly evaluated. This task was achieved by implementing a conformal prediction algorithm that enabled us to calculate robust prediction intervals for daily predicted case counts after May 2022. We observed that the reported case counts from the university EHR database consistently fell below the lower bound of the 95% conformal prediction intervals (Figure S3). These results suggest that accounting for the sampling uncertainty, reported daily case counts are consistently lower than would be expected based on measured SARS‐CoV‐2 RNA concentrations in the collected wastewater samples, providing further evidence of systemic COVID‐19 case count underreporting.

There are several limitations of this study. First, prediction model provides a smooth prediction trajectory of daily case counts and is unfortunately unable to accommodate sharp spikes in the observed case counts at times such as big social gathering (e.g., university football games) and the beginning of new semesters. Also, the study population is restricted to the University of Michigan campus, which may limit the generalizability of the findings to broader populations. Dormitories represent a narrow age cohort (17–25 years) with lower rates of severe COVID‐19 symptoms compared to older populations. Furthermore, while dorms are ideal for studying clustered outbreaks (e.g., 45–200 residents per dorm), their closed populations differ from open communities with variable mobility. In addition, the chosen timeframe skews signals due to varying occupancy and viral shedding patterns over semesters. For example, winter breaks or summer semesters have low occupancy. Finally, the reliability of our results is predicated on the accuracy of the U‐M daily clinical case counts up to May 2022, given that these data were used to construct the PLSR prediction model. This timeframe was selected since it preceded the cessation of the public transportation mask mandate on April 18, 2022 in the city of Ann Arbor, MI, USA, and coincided with a period of adequate viral testing rates, thus ensuring these reported case count data to be accurately aligned with the true COVID‐19 case counts during this period. Although this assumption is likely reasonable, it cannot be verified with absolute certainty. The in‐sample prediction performance of the models would be severely harmed if the data collected before May 2022 were found to be inaccurate, though Figure S1 suggests that this is not the case.

Despite these limitations, this university‐based community monitoring study clearly demonstrates the power of machine learning prediction analyses through the wastewater‐based surveillance to deliver a more accurate trajectory of COVID‐19 case counts for the university community, particularly in the context of widespread underreporting in a high‐mobility population. Future research could explore the application of this approach to other pathogens and consider the inclusion of additional predictors (e.g., social events) to enhance prediction accuracy. The ability to provide a more accurate estimates of true case counts can inform more effective public health policies and interventions, ultimately aiding in the control of disease spread.

In conclusion, this study demonstrates the critical role of machine learning in wastewater‐based epidemiology to address the challenges of underreporting in infectious disease monitoring. The approach presented here may be of use to governments and other public health practitioners interested in studying community‐level monitoring of disease spread dynamics when testing is either unavailable or adherence to testing protocols is low. With more accurate case count data, government policymakers can implement public health policies that better reflect the true spread of diseases of interest and understand potential risk of post‐COVID‐19 condition. Furthermore, these results are broadly applicable to areas in which sewage systems are present across different communities. It is worthing noting that our study did not impose community‐specific assumptions, so other regions with similar wastewater‐based surveillance programs may apply our approach to study underreporting.

## AUTHOR CONTRIBUTIONS

**Nathan Szeto**: Formal analysis; investigation; writing—original draft; writing—review and editing. **Jianfeng Wu**: Data curation; investigation; methodology; project administration; writing—original draft; writing—review and editing. **Yili Wang**: Data curation; formal analysis; investigation; methodology; visualization; writing—original draft; writing—review and editing. **Xin Li**: Data curation; investigation; methodology; writing—original draft; writing—review and editing. **Zheshi Zheng**: Formal analysis; investigation; writing—original draft; writing—review and editing. **Leyao Zhang**: Data curation; investigation; methodology. **Richard Neitzel**: funding acquisition; project administration; supervision. **Marisa Eisenberg**: Conceptualization; funding acquisition; investigation; methodology; project administration; supervision. **J. Tim Dvonch**: Funding acquisition; project administration; supervision. **Alfred Franzblau**: Conceptualization; methodology; project administration; supervision. **Peter X. K. Song**: Conceptualization; formal analysis; funding acquisition; investigation; methodology; project administration; software; supervision; visualization; writing—original draft; writing—review and editing. **Chuanwu Xi**: Conceptualization; funding acquisition; investigation; methodology; project administration; supervision; writing—original draft; writing—review and editing.

## ETHICS STATEMENT

The environmental epidemiology study considered in this study does not involve patients. There is no patient consent required in this study as no data are collected from any patients.

## CONFLICT OF INTERESTS

The authors declare no conflict of interests.

## Supporting information

Supporting Information.

## Data Availability

The data supporting the findings of this study are available from the corresponding author upon request.
