# Supplementary material for: Community‐level wastewater surveillance with machine learning methods to assess underreporting of COVID‐19 case counts
Source: mLife. 2025 Dec 3;4(6):717–20. doi: 10.1002/mlf2.70055 (PMC12754624; doi:10.1002/mlf2.70055)
Supplement: Supplementary file 1 — Supporting Information. [file MLF2-4-717-s001.docx]

# Materials and methods

1. *Wastewater sample collection*

At the University of Michigan, nine on-campus manholes were systematically selected for sampling, corresponding to seven residence halls and two townhouse areas that accommodate approximately 60% of the on-campus dormitory population. The selection of these sites was based on an in-depth review of the building blueprints and plumbing infrastructure. Each chosen manhole was specifically designated to have only one input source, thereby ensuring that collected wastewater came exclusively from the entire population of the respective residence halls.

Wastewater samples were collected once every weekday, except when extreme weather conditions were present (e.g., heavy rain and snow from September 2021-April 2023). Previous work has suggested that environmental factors such as rainfall are unlikely to significantly affect monitoring results generated from wastewater samples^1^.

ISCO GLS compact autosamplers (GLS, Teledyne ISCO, Lincoln, NE) were used for the composite sample collection from seven residence halls. Wastewater samples were taken over a 24-hour period at 15-minute intervals and 50 mL of wastewater was collected each time. Grab samples were taken from two townhouse areas, where autosamplers could not be installed. An extendable pole with sterile water sample bottles were used for grab wastewater sample collection. In addition, a 100 mL wastewater sample was taken each time at 5-minute intervals. Finally, 500 mL wastewater samples were collected at every sampling site.

2. *Wastewater sample processing*

A 50 mL aliquot of each sample was centrifuged at 3,500 rpm for 15 minutes at 4°C, followed by filtration through a 0.2 μm filtering unit (ThermoFisher, Ann Arbor, MI) to remove cellular material. Then, aliquots were concentrated using the PEG precipitation method^2^ Viral RNA was extracted using a QIAamp Viral RNA Mini Kit (Cat. # 52904. Qiagen, Valencia, CA) according to the manufacturer’s instructions. RNA extracts were stored in -80℃ for later use.

3. *Quantification of SARS-CoV-2 in samples*

The One-step transcriptase-polymerase chain reaction (RT-qPCR) method targeting the N1 and N2 region was used to quantify SARS-CoV-2 RNA concentrations following the method published by the US CDC^3^. The primers and probes used in the One-step RT-qPCR assay are summarized in supplementary Table S.1. A synthetic RNA of SARS-CoV-2 virus, purchased from ATCC (ATCC® VR-3276SD™, including fragments from ORF 1ab, Envelope, and Nucleocapsid regions) was used as a positive control and standard. All samples were run in duplicate. For genome copy quantification, a standard curve was generated with synthetic SARS-CoV-2 RNA. A series of 10-fold serial dilutions of the positive control, with concentrations ranging from 10,000 to 1 copy per µL, was used for each RT-qPCR plate.

The limit of detection (LoD) of the assay and method was determined by following previously published methods^4^. In practice, each RT-qPCR plate had duplicates of standards and the lowest concentration at which all the replicates were positive was considered as the LoD of the RT-qPCR plate. For quality control, both the extraction blank and reagent blank were also included in each RT-qPCR plate to identify any carryover contamination and inhibition.

### Table S.1. SARS CoV-2 and Phi6 primer and probe set particulars

| **Target** | **Primer/Probe name** | **Primer/Probe Sequence** | **Reference** |
| --- | --- | --- | --- |
| SARS CoV-2 | 2019-nCoV_N1-F  2019-nCoV_N1-R  2019-nCoV_N1-P | 5’-GACCCCAAAATCAGCGAAAT-3’  5’-TCTGGTTACTGCCAGTTGAATCTG-3’  5’-FAM-ACCCCGCATTACGTTTGGTGGACC-BHQ1-3’ | 3 |
|  | 2019-nCoV_N2-F  2019-nCoV_N2-R  2019-nCoV_N2-P | 5’-TTACAAACATTGGCCGCAAA-3’  5’-GCGCGACATTCCGAAGAA-3’  5’-HEX-ACAATTTGCCCCCAGCGCTTCAG-BHQ1-3’ | 3 |
| Phi6 | Φ6Tfor  Φ6Trev  Φ6Tprobe | 5’-TGGCGGCGGTCAAGAGC-3’  5’-GGATGATTCTCCAGAAGCTGCTG-3’  5’- FAM-CGGTCGTCGCAGGTCTGACACTCGC-BHQ1-3’ | 5 |

**Note on Probe Optimization:** If background signal seems high (negative droplets), Bio-Rad recommends using an Iowa Black Quencher in lieu of the Black Hole Quencher.

## 4. Datasets and statistical model development

Data were collected from two data sources. The first set of data consisted of daily concentrations of SARS-CoV-2 RNA collected as outlined in the Methods sections 1-3. Linear interpolation was used to produce average daily concentration RNA quantities and to impute other missing RNA values. Linear interpolation has been widely used for imputing missing values for time series data and shown to perform well in comparison to other imputation methods under similar circumstances^6^. The second set of data consisted of COVID-19 case count data collected from the U-M Ann Arbor Campus and Community Public Health Dashboard (healthresponse.umich.edu/dashboard/). As mentioned previously, SARS-CoV-2 RNA quantities are measured for both the N1 and N2 viral genes. The RNA quantities from both rival genes were used when constructing the models.

Data from the period from August 21, 2021, to April 7, 2023, were selected for the analysis, while excluding dates corresponding to weekends and Thanksgiving, Winter, and Spring Breaks when activities levels on the university campus were highly atypical. The case count dataset also includes the specific locations from which the wastewater samples were collected and thus corresponding cases reported. Since our goal was to longitudinally predict overall COVID-19 cases, rather than cases by specific locations, the mean of SARS-CoV-2 RNA concentrations by day for the on-campus locations were taken as the overall SARS-CoV-2 RNA concentration for a given day. The two datasets were then merged with the excluded dates removed, resulting in a merged dataset with 521 observations. Standard normalization was performed by adding 1 to and taking the base-10 log of both case count and SARS-CoV-2 RNA concentration variables.

Finally, a linear transitional time-series model was employed to forecast daily confirmed COVID-19 case counts, utilizing both concurrent and 7-day lagged concentrations of SARS-CoV-2 RNA N1 and N2 sequences, denoted by $G_{1}\left( t-i \right)$ and $G_{2}\left( t-i \right)$, $i=1,...,7, t=1,\ldots,T.$ Specifically, we implemented, via the `plsr` function from the `spls` package in R, a partial least squares regression (PLSR) prediction model with all 3 latent components as RNA predictors, with the number of latent components selected via 10-fold cross-validation to minimize Root Mean Square Error of Prediction (RMSEP). In so doing, the autocorrelation between the N1 and N2 RNA measurement time series can be adjusted for in order to avoid multicollinearity issues. PLSR regression inherently introduces sparsity by removing unimportant predictors from the model, thereby improving interpretability and prediction accuracy. We also implemented other prediction models, including methods of neural network and random forests. However, we did not observe meaningful improvements in model performance (i.e., R^2^, RMSEP, etc.) relative to PLSR. This is because the viral signals provided by RNA concentrations are typically rather weak such that nonlinear relationships cannot be meaningfully manifested. Finally, a PLSR model is inherently more interpretable than either a neural network or random forests that require large sample sizes in model training. We therefore proceeded with PLSR given our sample size.

**Autocorrelation**

Autocorrelation function plots (see Figures S.5-7) were generated to demonstrate that there was strong evidence in the collected university community data that the real clinical case counts and SARS-CoV-2 RNA quantities for a given day were highly correlated with those from previous days. Specifically, we observed that real clinical case counts for a given day were significantly associated with those preceding it by up to 21 days. And we observed that N1 and N2 SARS-CoV-2 RNA quantities for a given day were significantly associated with those preceding it by at least 27 days. Therefore, a translational modeling approach using lagged predictors, such as that implemented here, was likely justified for these long-memory dependencies shown in the data.

## **Uncertainty Quantification**

In order to further validate the results, an uncertainty quantification analysis was performed. Uncertainty quantification of PLSR models is still an open problem in the statistical literature and there is no theoretically guaranteed approach to generate prediction intervals. Conformal prediction methods were proposed and implemented to generate prediction intervals for the predicted case count values. Conformal prediction requires cutting the full dataset into a training and calibration set. The model is built on the training set, while inference is made based on a subset of the calibration set. The data set was split on January 12, 2022, in order to include more similar data as the target in both the training and calibration sets.

The generated prediction intervals allow us to quantify the uncertainty of the selected data subset and of the fitted PLSR model, thus providing some level of confidence in the predictions. It was observed that the reported cases tend to correspond to the lower bound of the prediction intervals. This correspondence further suggests that COVID-19 case counts are subject to systemic underreporting.

Conformal prediction is a robust predictive inference method that gives prediction intervals for algorithms that only have point predictions. It is robust in the sense that the prediction interval will have valid coverage even if the specified model is not accurate. This robustness property is particularly useful in this case because the PLSR algorithm lacks theoretical guarantees for consistency in model selection.

## **Supplementary Figures**

*Figure S.1. Comparison of U-M real clinical and predicted cases in log_10_ scale from September 2021 to May 2022, demonstrating high accuracy of in-sample prediction.*

*
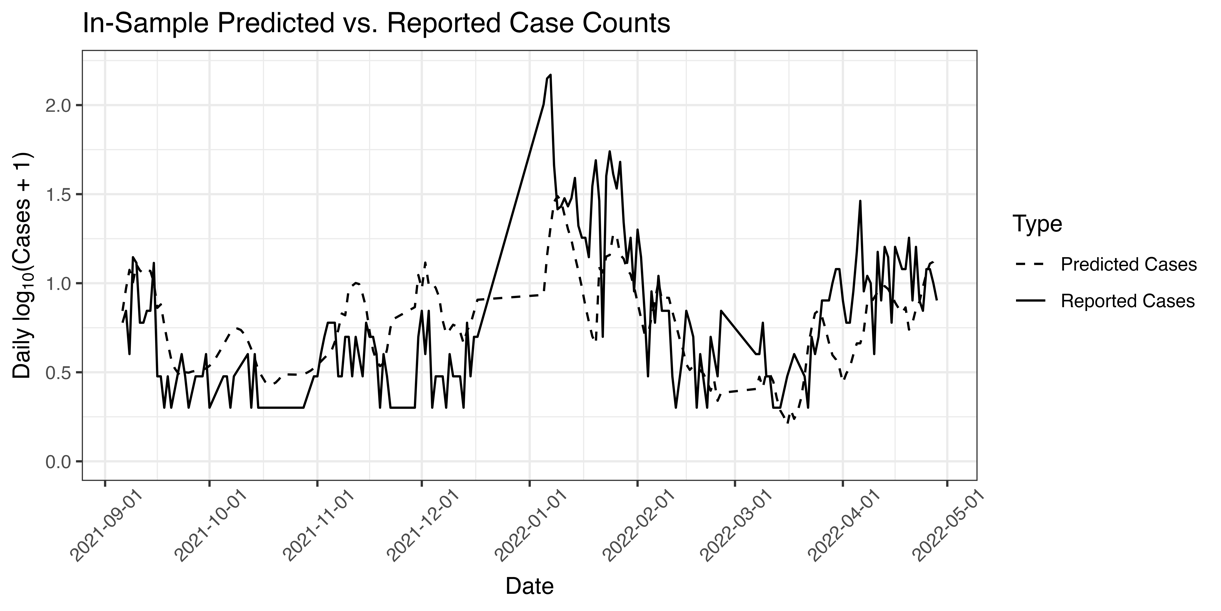
*

*Figure S.2. Comparison of community-level real clinical and predicted cases in log_10_ scale from May 2022 to April 2023, demonstrating systemic estimated underreporting of COVID-19 cases on the University of Michigan campus.*

*
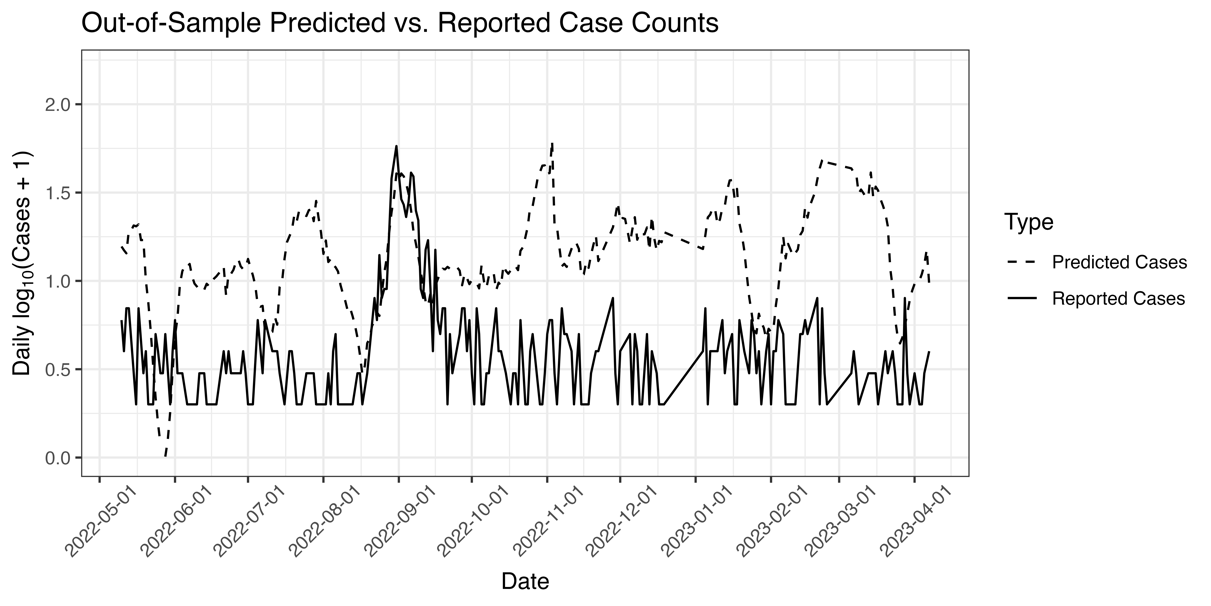
*

*Figure S.3. Reported daily case counts in log_10_ scale frequently fall around the 95% conformal lower predictive bound (CLPB) for the daily expected cases, demonstrating that reported daily case counts underestimate the expected number of cases in the university community.*


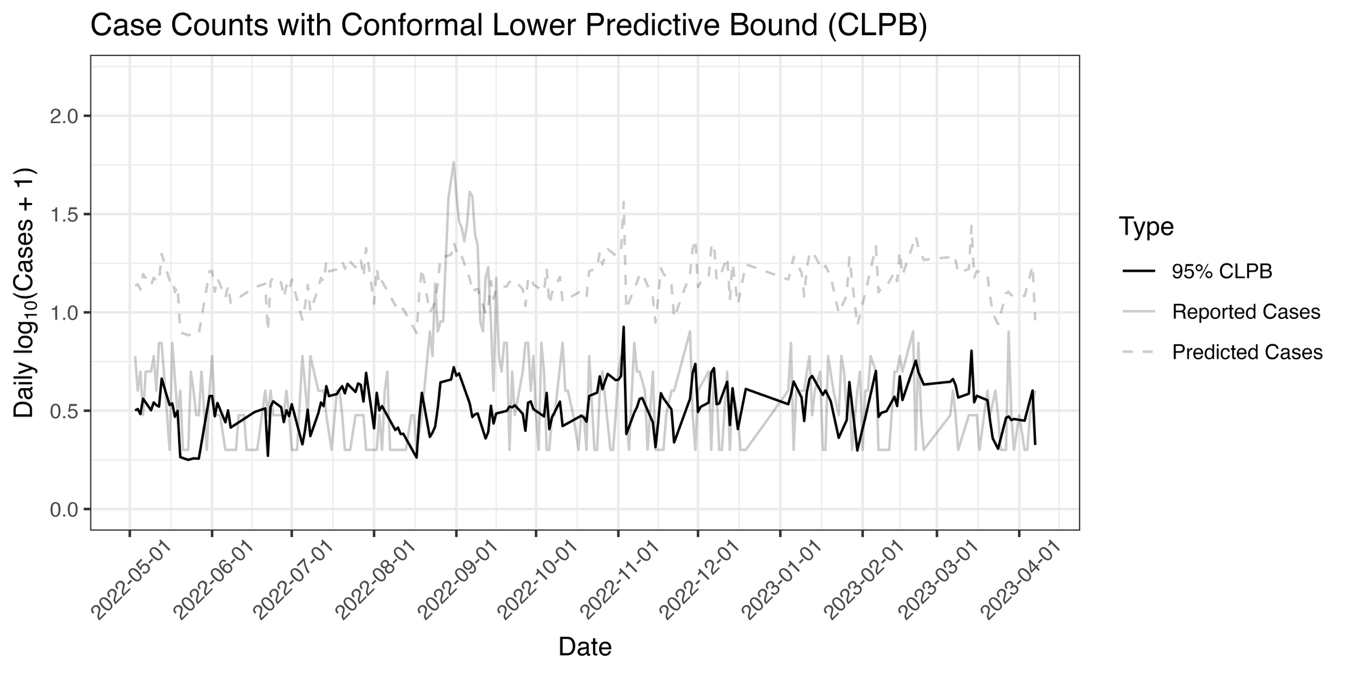


*Figure S.4. Distribution of underreporting magnitude post-May 2022. Negative values correspond to underprediction, positive values to underreporting.*


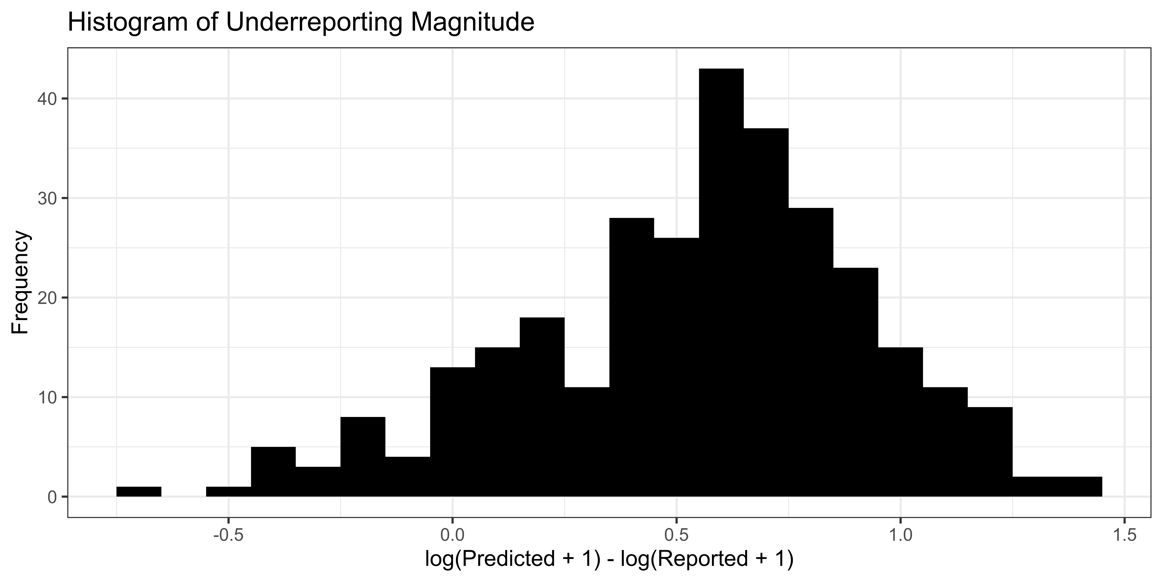


*Figure S.5. Autocorrelation function for daily real clinical case counts demonstrating that a significant autocorrelation for up to 21 days.*


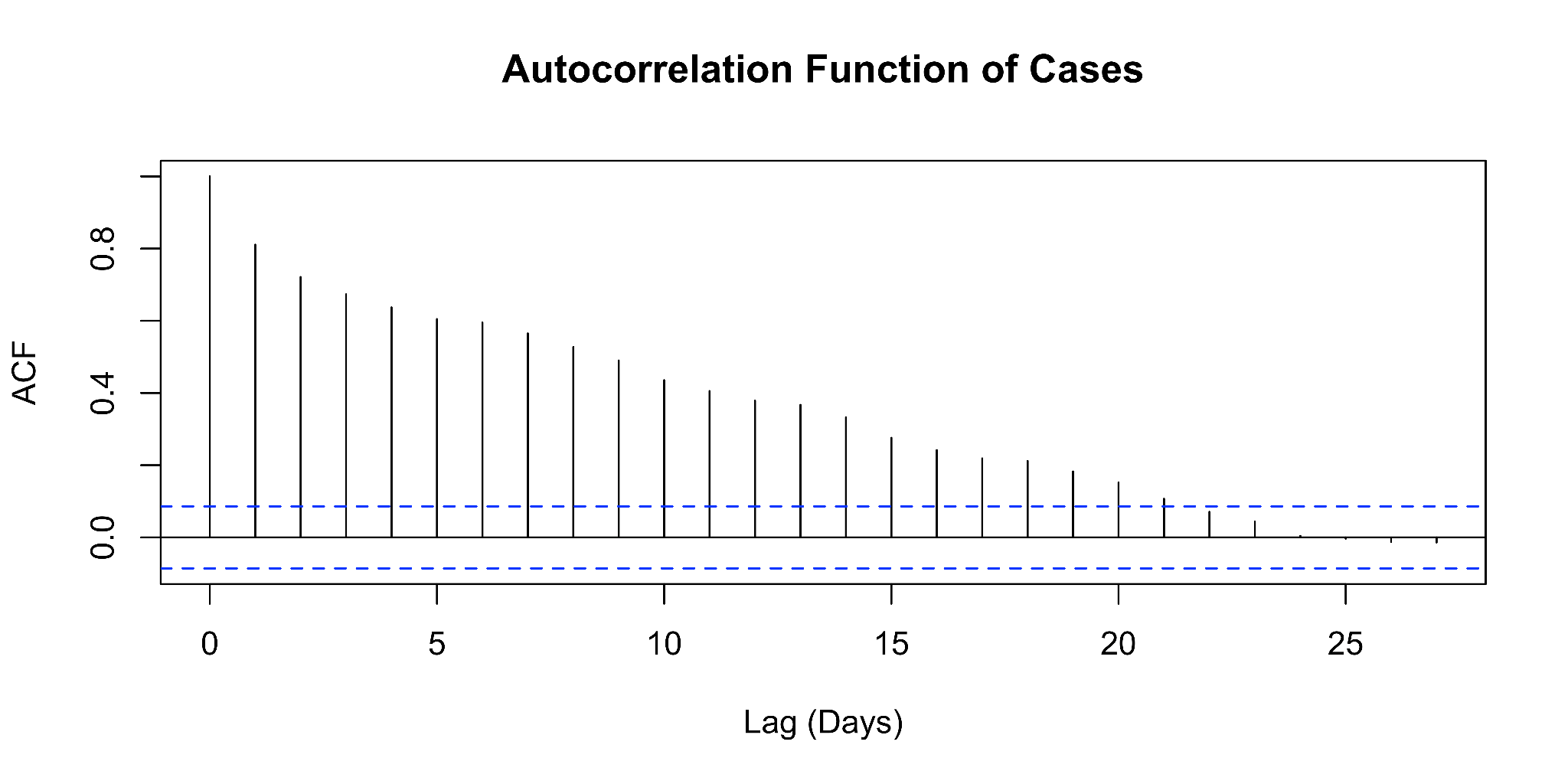


*Figure S.6. Autocorrelation function for N1 SARS-CoV-2 RNA quantities demonstrating a strong long-memory autocorrelation for at least 27 days.*


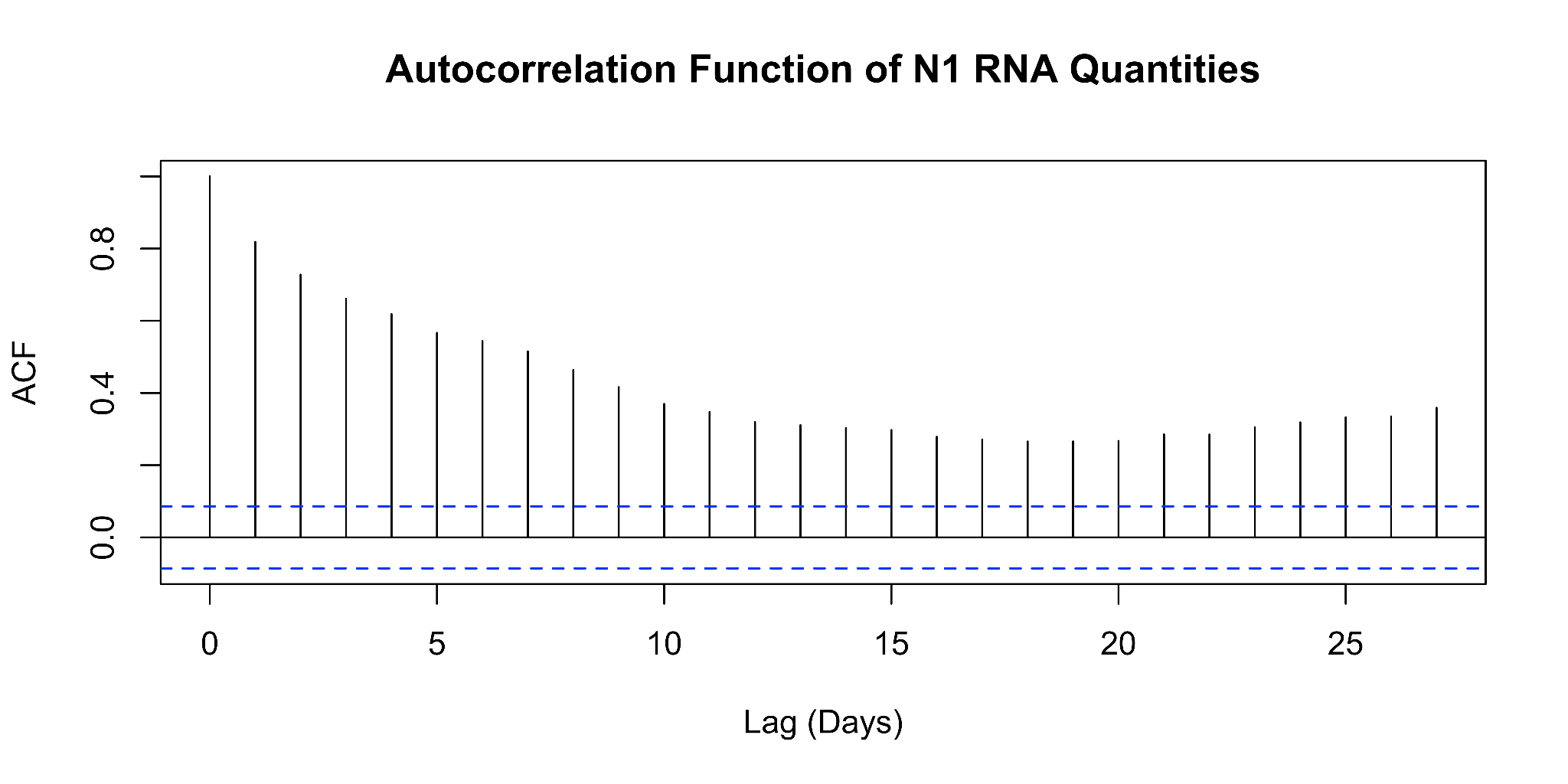


*Figure S.7. Autocorrelation function for N2 SARS-CoV-2 RNA quantities demonstrating a significant long-memory autocorrelation for at least 27 days.*


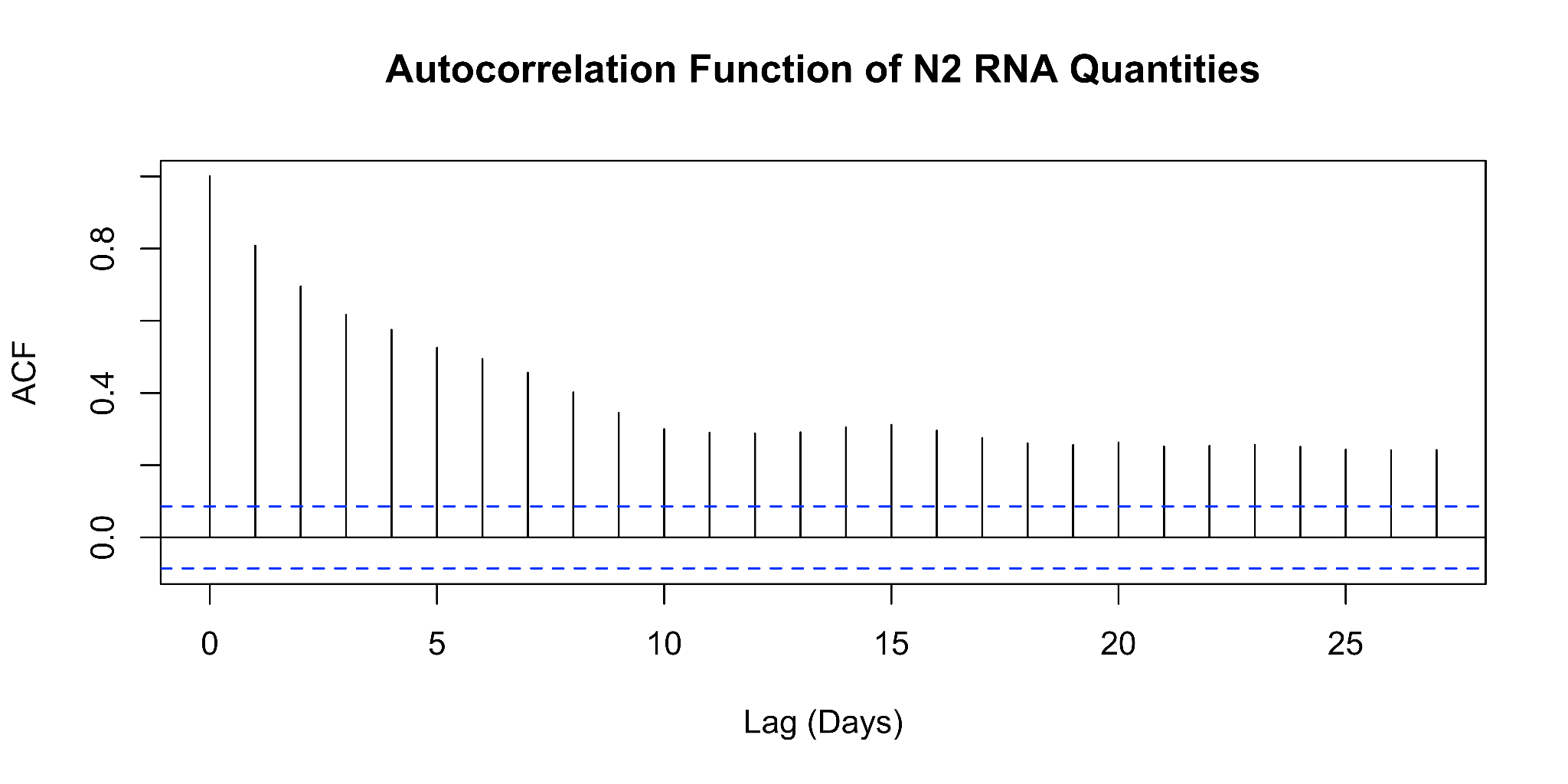


**References**

1. Triggiano F, De Giglio O, Apollonio F, Brigida S, Fasano F, Mancini P, et al. Wastewater-based epidemiology and SARS-CoV-2: variant trends in the Apulia region (Southern Italy) and effect of some environmental parameters. *Food Environ Virol*. 2023;15:331-41.
2. Wu F, Zhang J, Xiao A, Gu X, Lee WL, Armas F, et al. SARS-CoV-2 titers in wastewater are higher than expected from clinically confirmed cases. *mSystems*. 2020;5:e00614‐20.
3. National Center for Immunization and Respiratory Diseases (US), Division of Viral Diseases. 2019-novel coronavirus (2019-nCoV) real-time rRT-PCR panel primers and probes. Technical Report. Atlanta, GA: Centers for Disease Control and Prevention; 2020.
4. Bustin SA, Benes V, Garson J, Hellemans J, Huggett J, Kubista M, et al. The MIQE guidelines: minimum information for publication of quantitative real-time PCR experiments. *Clin Chem*. 2009;55:611-22.
5. Gendron L, Verreault D, Veillette M, Moineau S, Duchaine C. Evaluation of filters for the sampling and quantification of RNA phage aerosols. *Aerosol Sci Technol*. 2010;44:893-901.
6. Pham HT, Do T, Baek J, Nguyen CK, Pham QT, Nguyen HL, et al. Handling missing data in COVID-19 incidence estimation: secondary data analysis. *JMIR Public Health Surveill*. 2024;10:e53719.
